# Draft Genome Sequences of Endophytic *Pseudomonas* spp. Isolated from Grapevine Tissue and Antagonistic to Grapevine Trunk Disease Pathogens

**DOI:** 10.1128/MRA.00345-19

**Published:** 2019-06-27

**Authors:** Jennifer Niem, Regina Billones-Baaijens, Sandra Savocchia, Benjamin Stodart

**Affiliations:** a National Wine and Grape Industry Centre (NWGIC), Charles Sturt University, Wagga Wagga, NSW, Australia; b School of Agricultural and Wine Sciences, Charles Sturt University, Wagga Wagga, NSW, Australia; c Graham Centre for Agricultural Innovation (Charles Sturt University and NSW Department of Primary Industries), School of Agricultural and Wine Sciences, Charles Sturt University, Wagga Wagga, NSW, Australia; University of Arizona

## Abstract

Endophytic strains of Pseudomonas were isolated from grapevine tissues and exhibited antagonistic activity against several grapevine trunk disease pathogens. The draft genome sequences of the four strains revealed the presence of putative gene clusters that may impart biocontrol activity against plant pathogens.

## ANNOUNCEMENT

Species within Pseudomonas may be beneficial or detrimental to plant production systems. Efficacy has been established for Pseudomonas spp. as biocontrol agents against late blight and scab of potato (1, 2), Rhizoctonia root rot on bean (3), damping-off and root rot in tomato (4), black root rot of tobacco, and take-all disease of wheat (5, 6). In grapevines, Pseudomonas spp. are found in the phyllosphere (7–9) and inner tissues (10–12) and are known to suppress Botrytis cinerea (13, 14) and Rhizobium vitis (15).

*Pseudomonas* isolates BCA13, BCA14, and BCA17 were obtained from grapevine canes exhibiting Botryosphaeria dieback (BD) in Wagga Wagga, New South Wales (NSW), Australia. The canes were stripped of bark, surface sterilized, and placed on nutrient agar. Emerging bacteria were streaked onto King’s B medium to obtain single colonies. All isolates inhibited BD and Eutypa dieback (ED) pathogens in culture and reduced BD infection *in planta* (our unpublished data). A fourth isolate, JMN1, was obtained from an asymptomatic vine in Harden (NSW, Australia) by suspending internal trunk wood shavings in Ringer’s solution. Single colonies were selected by streaking on King’s B medium at 25°C. JMN1 was not antagonistic to BD and ED pathogens. The four isolates were identified by amplification and sequencing of the 16S rRNA and *rpoD* genes. Gene sequences were subjected to BLASTn searches of the NCBI database, and reference sequences were selected for phylogenetic analyses. Sequence alignment was completed with Clustal W, and a neighbor-joining tree was constructed within MEGA 7 (16). The four isolates were found to be closely related to Pseudomonas poae.

Each isolate was grown in nutrient broth for 24 h at 25°C and then harvested for DNA extraction using the Gentra Puregene bacterial DNA extraction kit (Qiagen), following the manufacturer’s specifications. Shotgun library preparation and Illumina sequencing (HiSeq 2500 platform) were conducted by the Australian Genome Research Facility, resulting in 12,569,718 reads (150-bp paired ends; Table 1). Data were generated with the Illumina bcl2fastq pipeline version 2.20.0.422. Draft genomes were assembled using the Unicycler assembler, implementing an optimizer for SPAdes 3.13.0 (17). *k-mer* lengths between 0.2 and 0.95 of total read length were examined, and contigs of <200 bases were removed. Annotation was completed with the NCBI Prokaryotic Genome Annotation Pipeline (PGAP) 4.7 (18) and the Rapid Annotations using Subsystems Technology server, implementing RAST*tk* (19). Default parameters for all software programs were used, unless otherwise specified.

**TABLE 1:** Genome information and accession numbers of four *Pseudomonas* strains isolated from grapevine tissue

Strain	SRA accession no.	No. of reads	Assembly size (bp)	No. of contigs	*N*_50_ value (bp)	G+C content (%)	No. of coding genes	No. of RAST subsystems represented
BCA13	SRX5463364	3,189,991	6,318,228	34	1,278,996	60.18	5,171	370
BCA14	SRX5463365	2,798,878	6,322,821	34	562,240	60.18	5,666	403
BCA17	SRX5463366	3,028,714	6,318,257	28	760,227	60.18	5,559	403
JMN1	SRX5463367	3,552,135	6,322,966	29	691,035	60.18	5,564	401

Gene clusters which may play a role in the control of plant pathogens were identified. Queries of the Plant-bacteria Interaction Factors Resource (PIFAR) (20) found that each strain of *Pseudomonas* contains a remarkable number of putative biocontrol gene clusters, including those responsible for lipopeptide antibiotics, siderophores, proteases, detoxification, lipopolysaccharides, multidrug resistance, microbe-associated molecular proteins (MAMPs), and biofilms. antiSMASH 4.0 (21) was implemented to detect gene clusters responsible for the biosynthesis of secondary metabolites, resulting in the identification of clusters containing nonribosomal peptide synthetases with known activity as antimicrobial agents, including poaeamide, rhizomide, and rhizoxins.

### Data availability.

The genome sequences for BCA13, BCA14, BCA17, and JMN1 are available under NCBI BioProject number PRJNA522029, with annotated assemblies available under accession numbers SGWK00000000, SGWJ00000000, SGWI00000000, and SGWH00000000, respectively. The Sequence Read Archive (SRA) accession numbers are listed in Table 1. Sequence reads were deposited in the NCBI SRA under the accession numbers SRR8667294 to SRR8667297.
